# Technology-enabled Behavioral Health Integration Decreases Emergency Department Utilization

**DOI:** 10.2174/17450179-v18-e2208150

**Published:** 2022-09-20

**Authors:** Adam Pardes, Rachelle Rene, Phansy Chun, Mollie Cherson

**Affiliations:** 1 NeuroFlow, Inc., Philadelphia, PA, United States; 2 Jefferson Health, Thomas Jefferson University, Philadelphia, PA 19144, United States

**Keywords:** Behavioral health, Technology, Integrated care, Anxiety, Depression, Mental health care

## Abstract

**Background:**

Behavioral health integration allows for patient-centered care, leads to higher levels of provider-patient engagement, and is key to improving patient outcomes. However, behavioral health integration is administratively burdensome and therefore is often not adopted. Technology presents opportunities to increase care team efficiency and improve patient outcomes. The goal of this study was to retrospectively compare clinical outcomes and emergency department utilization in patients using a technology platform compared to patients receiving treatment as usual.

**Methods:**

The technology platform, NeuroFlow, was deployed to deliver technology-enabled behavioral health integration in 30 clinics, and 598 electronic health records were analyzed.

**Results:**

In the six-month period following technology-enabled behavioral health integration implementation, emergency department utilization decreased by 34% in the treatment group (n=259), while increasing by 58% in the treatment as usual group (n=339). Additionally, statistically significant (p < .01) decreases in PHQ-9 (-17.3%) and GAD-7 (-12.4%) scores were only observed in the treatment group.

**Conclusion:**

Findings from this study support use of a technology-enabled behavioral health tool to decrease emergency department use and highlight the importance of measurement-based care. Future research will be key to enhancing behavioral health technology and integration to further improve patient outcomes and reduce emergency department utilization.

## 1 INTRODUCTION

Systematic decentralization, or the transition of mental health care and treatment from mental health hospitals to primary care and community settings, has long been emphasized by the World Health Organization (WHO) with a goal of integrating mental health services into primary care across 80% of countries by 2030 [1]. The results of behavioral health integration, such as collaborative psychiatric care, have demonstrated improved patient outcomes and lower total cost of care [2].

Notably, Italy remains the only country in the world to have abolished psychiatric hospitals for community-centered care. In addition to the human rights benefits, both patients and staff report being satisfied with the community-based services. During the COVID pandemic, patient and staff satisfaction with care remained high, while non-psychiatric healthcare workers expressed dissatisfaction with increased rates of provider burnout and related mental health conditions [3]. The city of Trieste, Italy, transitioned to a fully community-based model in 1980 and has been recognized by the WHO for innovative approaches to psychiatric care, including the use of innovative mental health technologies for interactive psychiatric consultation and video conferencing [4]. Additional mental health technologies, including virtual reality and internet-based interventions, have additionally been effectively deployed in the community setting in Italy [5, 6].

Despite the outcome and cost benefits, behavioral health integration (BHI) is a complex process and has not been widely adopted [7]. BHI combines care in one setting for medical conditions and behavioral and mental health, with an aim of providing whole-person care [8]. BHI allows for patient centered care, supports the efforts of primary care physicians, and leads to higher levels of provider-patient engagement [9]. However, more research is needed to determine if BHI implementation programs can scale with large practices, thereby reducing administrative burden and increasing the reach of the care teams. Additionally, for integrative digital platforms to be accepted into the provider’s office, the platforms must be adaptable for different patient populations, such as pregnant and postpartum women [10, 11].

One utilization metric to determine the quality of care management and access to BHI services is emergency department (ED) visits. Many ED visits can be prevented or treated in primary care or other lower levels of care [12]. Untreated and undertreated behavioral health conditions also may lead to worsening physical health conditions [13]. Better management of mental health conditions in primary care may lead to decreased ED utilization [12]. This has become increasingly relevant, with the proportion of ED visits related to behavioral health conditions increasing from 6.6% to 10.9% between 2007-2016 [14]. This has only escalated during the COVID-19 pandemic, with even higher rates of mental health-related ED visits, particularly among young people [15, 16].

While there has been increased adoption and expanded use of mental health technologies during the pandemic to address this crisis, technology remains underutilized. Self-management solutions alone (i.e., mental health apps) have limited evidence, but mental health technology solutions that seek to augment and extend the mental health workforce have the unlimited potential [15]. The pandemic provides an opportunity to encourage mental health technology adoption and innovation. Programs should be customized to each patient to meet their treatment plan goals, and staff should be equipped with the skills to make informed clinical decisions with the tools [16]. An evaluation framework should be used when making a decision to implement a mental health technology program [17-19].

The aim of this study was to assess the effects of technology-enabled behavioral health integration (tBHI) programs on emergency department (ED) utilization and clinical outcomes in a large urban health system. We conducted an analysis of patients enrolled in a tBHI program compared to those receiving traditional psychotherapy and medical services (TAU) to determine if there was an effect on ED utilization as measured by EHR data review. Secondary analysis compared the clinical outcomes of the two groups as measured by the Patient Health Questionnaire (PHQ-2/9) and Generalized Anxiety Disorder-7 (GAD-2/7).

## 2 MATERIALS AND METHODS

NeuroFlow’s technology platform was deployed in 30 clinics, including primary care (27 clinics), obstetrics and gynecology (OBGYN, 2 clinics), and psychiatry (1 clinic; not included in this study), at a large urban health system (Jefferson Health). The NeuroFlow platform is a HIPAA-compliant, cloud-based tool that facilitates behavioral health access, engagement and remote measurement-based care. The platform offers both a patient-facing mobile application with an accompanying clinician-facing web platform that provides clinical decision support. The technology was used to remotely administer and collect more than 6,200 validated assessments from patients, both PHQ-2/9s and GAD-2/7s delivered monthly, in accordance with study procedures. Based on the results of these assessments, the technology also automated the assignment of suggested psychoeducation and self-care activities in the mobile app. The tBHI program varied by specialty. Embedded behavioral health consultants (BHCs) were in primary care clinics as part of the behavioral health integration requirements of the Comprehensive Primary Care Plus (CPC+) model developed by the Centers for Medicare and Medicaid Services (CMS) Innovation Center [20, 21]. The BHCs monitored the platform for increased severity or risk of suicide daily and were able to assign personalized patient homework that patients could complete remotely in the NeuroFlow app. The OBGYN tBHI program was designed for pregnant women and new mothers susceptible to perinatal mood and anxiety disorders. Referring providers reviewed monthly patient status reports generated by NeuroFlow. Medical assistants supported with enrollment, patient education, and reviewing the platform for increased condition severity or risk of suicide daily in the OBGYN clinics.

In this retrospective study, the Jefferson Health data team reviewed electronic health record (EHR) data from 598 patients in clinics with tBHI programs. Patients were divided into different treatment groups, TAU or tBHI. Patients were eligible if they had a documented F-code (behavioral health) diagnosis and were receiving primary care services at a clinic with an established tBHI program, and had an encounter within ±60 days of the mid-point of the study period. The tBHI program was implemented in May, 2019. The study period was six months pre- and six months post tBHI implementation from November 1^st^, 2018 to November 30^th^, 2019.

The independent variable in this study was the use of tBHI technology, with the primary dependent variable being the number of emergency department visits. This was measured and determined *via* chart review. We hypothesized that using tBHI technology for remote measurement-based care, patient engagement, and clinical decision support would decrease the number of ED visits relative to TAU.

Patients that consented to tBHI services and registered for the NeuroFlow application were enrolled in the tBHI arm of the study. Due to the retrospective nature of this study, a control group of TAU patients were identified prior to data analysis by a comprehensive chart review and were subjected to rigorous exclusion criterion to ensure accurate matching with the tBHI population. Specifically, TAU and tBHI patients were only included in the analysis if they had ambulatory encounters at least 90 days before and after the technology’s deployment, had an F-code (behavioral health) diagnosis, and had at least two PHQ-2/9 and/or GAD-2/7 scores in the EHR. Only participants 18 years and older were included in the study. Ambulatory visits included hospitalizations, outpatient encounters, inpatient encounters, or visits for labs. In the subset OBGYN population analysis, inclusion criteria were pregnant women, using diagnosis code Z34.90 and who had an encounter before and after the technology’s deployment.

Data were analyzed as the number of patients who had ambulatory encounters in the EHR six months before tBHI implementation (November, 2018 – May, 2019) as compared to six months following the deployment of tBHI (May – November, 2019). The number of emergency department visits in the EHR was then normalized based on the sample size, and percent change pre- and post-tBHI introduction was measured. Data were analyzed using a student’s t-test. For all tests, the significance level was set at p < 0.05. n refers to the number of patients in a given group.

## 3 RESULTS

**Fig. (1):**
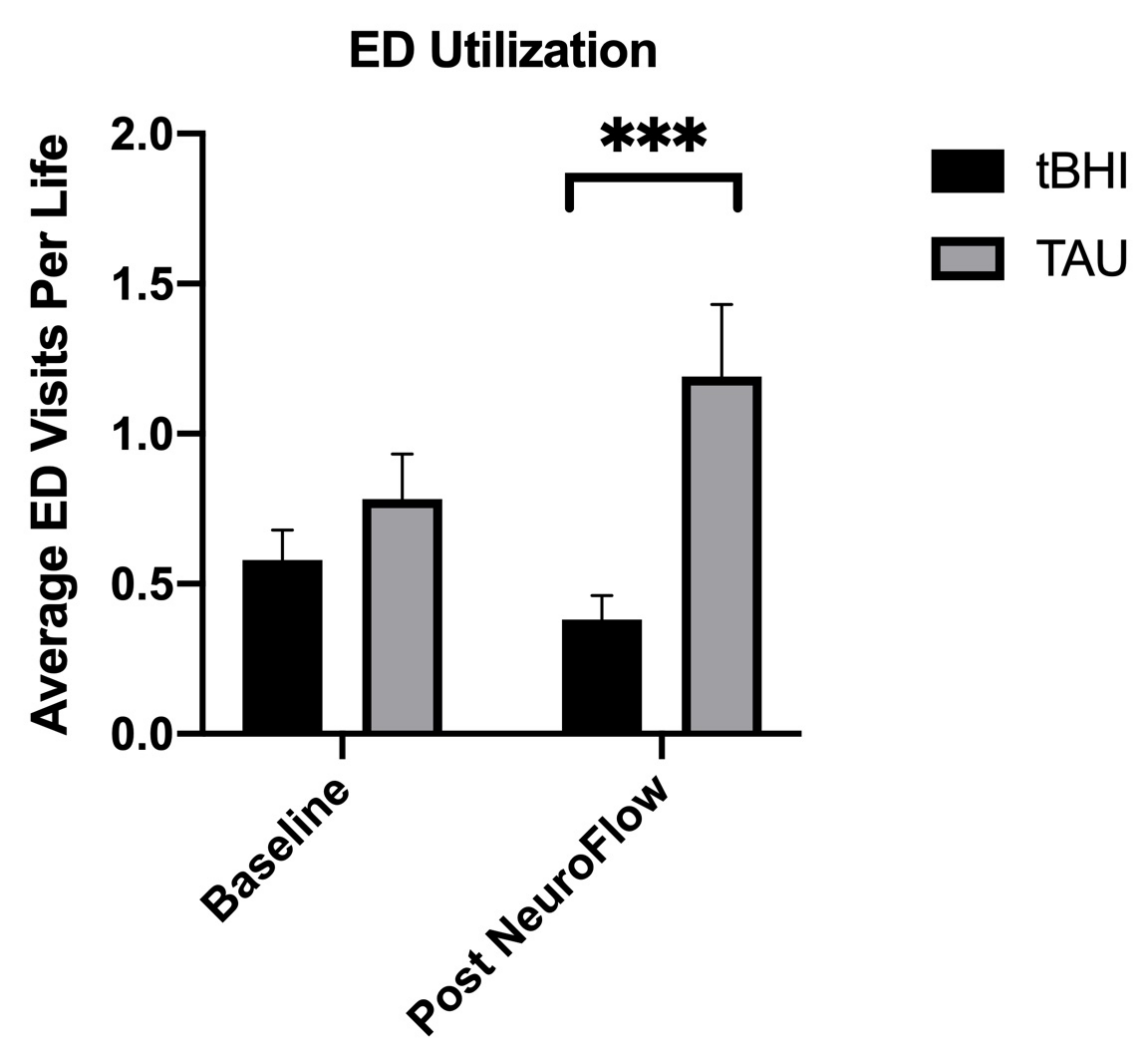
Emergency department (ED) utilization decreased significantly six months after using the NeuroFlow tBHI platform.

In the six months prior to deploying the technology (baseline), the tBHI (n = 259) and TAU (n = 339) groups had similar ED utilization of 0.58 (SD = +/-1.660) and 0.78 (SD = +/-2.78) visits per life, respectively. In the six-month period post-technology implementation, ED utilization decreased by 34% in the tBHI group while concurrently increasing by 52.5% in the TAU group. This finding indicates significantly less ED utilization in the tBHI group that used technology as compared to the TAU group (p = 0.002) (Fig. **1**). The tBHI group also yielded promising results based on reductions in symptoms of anxiety and depression. For instance, statistically significant decreases compared to baseline were found in scores on both the PHQ-9 (-17.3%, p = 0.01) and the GAD-7 (-12.4%, p < 0.001) from tBHI participants. In contrast, those differences were not observed in the TAU group. Overall, 82% of patients that utilized technology reported symptom reduction for depression, and 77% reported symptom improvement for anxiety according to these validated scales. The tBHI group additionally had a 19% higher assessment completion rate compared to the TAU group with EHR alone.

To analyze how the technology platform can be used in different patient populations, custom OBGYN modules and journeys providing educational content were created for perinatal mood and anxiety disorders, such as postpartum depression. Utilizing inclusion criteria similar to the main group (adjusted for pregnancy), a separate analysis was completed evaluating tBHI (n = 42) and TAU (n = 28) in OBGYN patients. In this smaller cohort, nearly identical results were found as ED utilization decreased by 35% compared to baseline in the tBHI group, whereas it increased by 46% compared to baseline in the TAU group.

No covariates were used in the analysis, as this study reviewed EMR data to evaluate the effectiveness of the tBHI program in reducing ED utilization.

## 4 DISCUSSION

The goal of this study was to compare clinical outcomes and emergency department (ED) utilization rates in patients using a technology-enabled behavioral health integration (tBHI) platform as compared to those receiving treatment as usual (TAU). The study findings highlight how deploying a tBHI tool with patients can increase screening rates and help decrease ED utilization.

A primary barrier to integrating BHI into primary care is the effectiveness of remotely deploying behavioral health assessments [11, 21]. The results from this study demonstrated that there was higher compliance with mobile technology compared to TAU. Additionally, statistically significant decreases in PHQ-9 and GAD-7 scores were only observed in the tBHI group, which may have been impacted by more data-driven clinical decision support by the embedded BHCs and/or the personalized exercises patients engaged within the NeuroFlow app. Further research will be key in identifying ways that behavioral health technology can further enhance patient outcomes. Further research should also address the analyses of covariates, including specific mental health diagnoses, prior hospitalizations, race/ethnicity, age, and the score of GAD-2/7 or PHQ-2/9. This study did not take any demographic data into analysis, and further research should investigate the impact of technology on different demographic groups.

A limitation of this study is that it is possible that participants in the study may have had ED visits that may not have been captured in the dataset. This may have occurred if participants went to a different ED outside of the participating health system. Another limitation is the nature of this design, as the retrospective cohort design can only show association and may be prone to recall bias. However, this study included a subgroup of patients to mitigate bias and improve confidence in the results. In an intentional effort to reduce any potential bias during the analysis, data about the specific behavioral health diagnoses in each sample and any other behavioral health care interventions (*e.g*., medication management, inpatient care, *etc*.) were not collected. The 34% decrease in ED utilization in the tBHI group may be accounted for by several aspects of the tBHI program. Of note, alerts were sent to providers if thoughts of self-harm were identified through assessment or journal activities. This also led to an immediate push of crisis resources to the patient. The 52.5% increase in ED utilization by the TAU group, in contrast, warrants additional investigation. Given that the study period did not overlap with the COVID-19 in March, 2020, this may be partially explained by the limitation of the retrospective nature of this study. Therefore, prospective randomized control studies are recommended to further evaluate the effects of a tBHI program. Additionally, further research is warranted to evaluate any condition-specific effects and impact on the utilization of higher levels of behavioral health care.

## CONCLUSION

Patients enrolled in a tBHI program that included remote behavioral health monitoring, digital activities, and clinical decision support for primary care and OBGYN providers significantly improved mental health outcomes and reduced ED utilization compared to TAU. This study suggests that technology to enhance integrated care programs may lead to a reduction in preventable ED utilization and, therefore, a reduction in the total cost of care.

## Data Availability

The datasets generated and analyzed for this study are available upon request.

## LIST OF ABBREVIATIONS

WHO: = World Health Organization
ED: = Emergency Department
EHR: = Electronic Health Record
tBHI: = Technology-enabled Behavioral Health Integration
